# The Role of Plastic Surgeons in Addressing Firearm Morbidity and Mortality

**DOI:** 10.7759/cureus.36414

**Published:** 2023-03-20

**Authors:** Rachel H Safeek, Jessica Ching, Harvey Chim, Ellen Satteson

**Affiliations:** 1 Plastic and Reconstructive Surgery, University of Florida College of Medicine, Gainesville, USA

**Keywords:** gun violence, firearm violence, gunshot wound, reconstructive surgery, plastic surgery

## Abstract

Firearm injuries are now the leading cause of pediatric mortality in the United States. With the number of firearm injuries increasing at an alarming rate, the American Medical Association (AMA) declared firearm violence a public health crisis. In response to this emerging public health issue, the American College of Surgeons (ACS) developed the STOP THE BLEED training to educate laypersons on how to mitigate acute hemorrhage following gunshot wounds (GSWs) and other ballistic injuries. Stabilization of patients following GSWs is often handled by a multidisciplinary team of trauma and reconstructive surgeons. Here, we describe the history and ongoing role of reconstructive surgeons in preventing and addressing firearm morbidity and mortality. Hand surgeons are uniquely positioned to counsel patients on firearm safety, e.g., educating patients on proper firearm storage away from minors in the home, in an effort to mitigate accidental firearm injury to the upper extremity. As the evolving climate of firearm violence continues to rise, plastic and reconstructive surgeons will continue to play a critical role in restoring form and function among patients afflicted with GSWs.

## Introduction and background

Since the start of the COVID-19 pandemic, firearm injuries, ranging from assault and suicide to accidental injury, have increased at an alarming rate over the last five years [1], with nearly 100,000 persons experiencing firearm injuries each year [2]. This epidemic has grown so steeply that, as of 2022, firearm injuries will be the leading cause of pediatric mortality in the United States [3,4]. In response to this emerging issue, the American Medical Association (AMA) declared firearm violence a public health crisis and highlighted the particular gravity of firearm injuries in the United States [5].

In 2018, the American College of Surgeons (ACS) developed STOP THE BLEED, a training course for non-healthcare personnel to mitigate acute hemorrhage in the event of a firearm injury [6]. The training offers the general public knowledge on how to correctly apply tourniquets and pack gunshot wounds (GSWs). Overall, surgeons play a critical role in addressing acute firearm injuries, with surgical management of GSWs involving a multidisciplinary team to stabilize any initial hemorrhage and trauma. Plastic and reconstructive surgeons remain an integral part of the process and may perform either the initial debridement and operative fixation of firearm injuries, e.g., GSWs to the extremities or head and neck, or become involved at a later time for the subsequent reconstruction of any disfigurement after the patient’s initial injuries have been stabilized by other surgical services.

## Review

Reconstruction of firearm injuries and the birth of plastic surgery

The role of modern plastic surgery in addressing firearm morbidity and mortality is evident from the origins of the specialty itself, which is rooted in the reconstruction of army-related injuries. With the advent of machine guns during World War I, firearm and ballistic injuries emerged as new surgical challenges. Facial injuries, in particular, increased during this period due to trench warfare, in which soldiers stood in man-made trenches, occasionally peering over parapets [7].

Ballistic injuries to the face were mostly due to firearm shrapnel, which could tear through flesh, annihilating skin and soft tissues. Often, these wounds were closed primarily on the front lines, as access to sterile operating rooms (ORs) was limited on the battlefield. In wounds with significant tissue loss, primary closures were problematic in that primary approximation of skin edges could lead to significant pulling of the skin tautly over the face. Following the wound healing stage, scar contracture would then further pull the skin, leaving patients disfigured and with functional limitations, including difficulty blinking, smiling, etc. Bony injuries of the nose could leave soldiers with a hole in their face, while injuries to the mandible could create significant difficulties with swallowing and chewing food, often putting soldiers at risk for aspiration [8,9].

In 1917, Harold Gillies, a British-trained New Zealand surgeon, developed the facial reconstructive techniques that laid the foundation for modern-day plastic surgery [10-12]. He established a facial wound ward at Cambridge Military Hospital, eventually opening The Queen’s Hospital, an entire hospital dedicated to facial injuries. Leveraging the fundamentals of skin grafting techniques of the time, Gillies harnessed skin pedicles from the adjacent skin of a wound to create skin flaps, which could be rotated from the donor site to cover large skin and soft tissue defects. The novelty of Gillies’ work was in his decision to maintain the connection of the pedicle to the donor site, preserving blood supply to the flap. This enhanced the wound healing process.

Gillies also proposed the "tubed pedicle," where the ends of the skin pedicle were sutured together in the shape of a tube. He found that, over time, this decreased the risk of infection and strengthened the overall blood supply to the flap. Later, the flap could be detached from its donor site and transferred to open wounds for coverage, which laid the foundation for modern plastic surgery [10-12].

Modern-day reconstruction

Extremity Injuries and Reconstruction

Firearm injuries to the upper extremity (UE) are among the most reported, with studies showing that among civilians, 70% of unintentional GSWs and 45% of assault-related GSWs involve the extremities [13,14]. A 2009 study showed that among 1622 GSWs in Detroit, 61.3% involved the extremities, with half (32.2%) involving the UE [15]. They are often the result of accidental injury, e.g., while cleaning, handling, loading, or unloading a firearm at home [16-18], as well as assault. Injuries can be classified as high-velocity, which are larger, more extensive injuries usually from long-barreled firearms, e.g., rifles, versus low-velocity injuries, which are usually from handguns and yield less extensive injuries [19].

Given the compact space of the upper extremity, there is a high concern for complex neurovascular injury with GSWs in this region. While most injuries are low-velocity and not typically fatal, emergent action is required to stabilize any ongoing hemorrhage and assess for signs of neurovascular damage, including ruling out compartment syndrome [20]. Operative management is typically focused on early debridement, repair of injured structures, operative fixation of any bony injuries, and soft tissue coverage.

For hand and upper extremity injuries sparing the bone, Allen’s test is performed to assess collateral blood flow to the hand [21]. Two-point discrimination is also tested for any signs of nerve damage. Compartment syndrome is identified by screening for any signs of the five Ps: pain out of proportion to the injury, pulselessness, pallor, paresthesia, and poikilothermia. When there is a concern for compartment syndrome or the potential for it to develop related to reperfusion injury following revascularization, fasciotomies are performed. In the forearm, this includes the release of the flexor compartments (deep and superficial), as well as the extensor and mobile wad of Henry compartments, typically through incisions in the volar and dorsal forearm. The hand consists of 10 total compartments: the thenar, hypothenar, adductor pollicis, four dorsal interossei, and three palmar interossei, which can be released through two dorsal hand incisions and incisions along the glabrous-non-glabrous junction on the radial and ulnar aspects of the hand.

**Figure 1:**
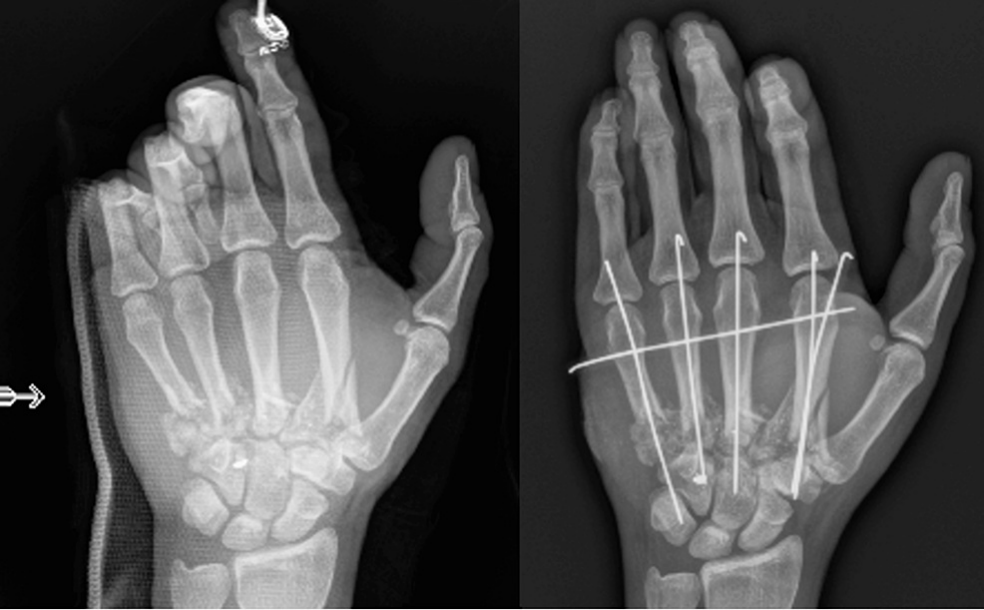
Hand gunshot wound pinned with K-wires for fracture stabilization. The figure is provided by the authors from our patient population. Informed consent has been obtained for the usage of the image.

Associated fractures are typically managed with non-operative immobilization, with or without closed reduction, or with surgical fixation based on the fracture pattern, associated injuries, and soft tissue coverage concerns. Operative management can include placement of an external fixator, internal fixation with a plate and screws, or insertion of percutaneous Kirschner wire (K-wire) pins to stabilize the bone and joint, with studies showing that nearly 41% of GSWs to the UE require K-wire placement [22] (Figure 1).

**Figure 2:**
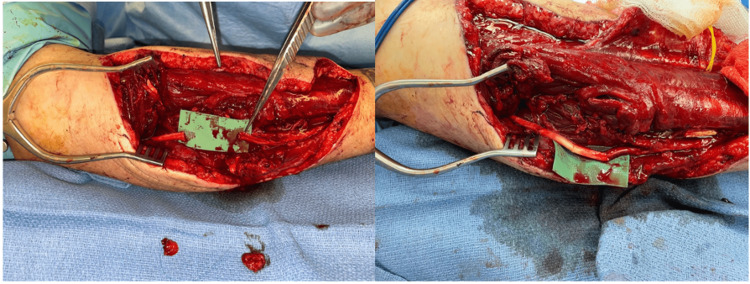
Ulnar nerve defect with nerve grafting and an end-to-side anterior interosseous nerve (AIN) to ulnar nerve transfer The figure is provided by the authors from our patient population. Informed consent has been obtained for the usage of the image.

For neurovascular injuries, microsurgical repair of blood vessels or nerves may be required. Often, the zone of injury from ballistic injuries is large, necessitating vein or nerve grafts to replace damaged segments of the artery or nerve, respectively. For more proximal nerve injuries, nerve or tendon transfers may help restore function when nerve regeneration may be inadequate or take too long to preserve motor endplate function (Figure 2).

**Figure 3:**
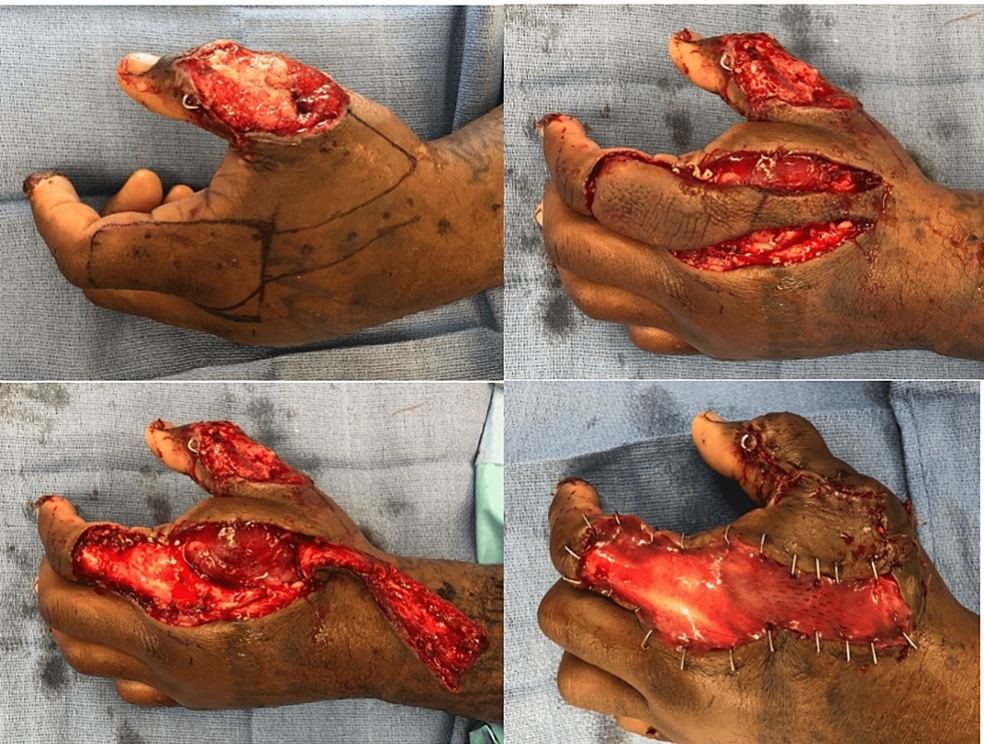
Gunshot wound to the upper extremity with forearm wound skin graft and thumb wound/open fracture that was pinned and covered with a first dorsal metacarpal artery (FDMA) flap. The figure is provided by the authors from our patient population. Informed consent has been obtained for the usage of the image.

**Figure 4:**
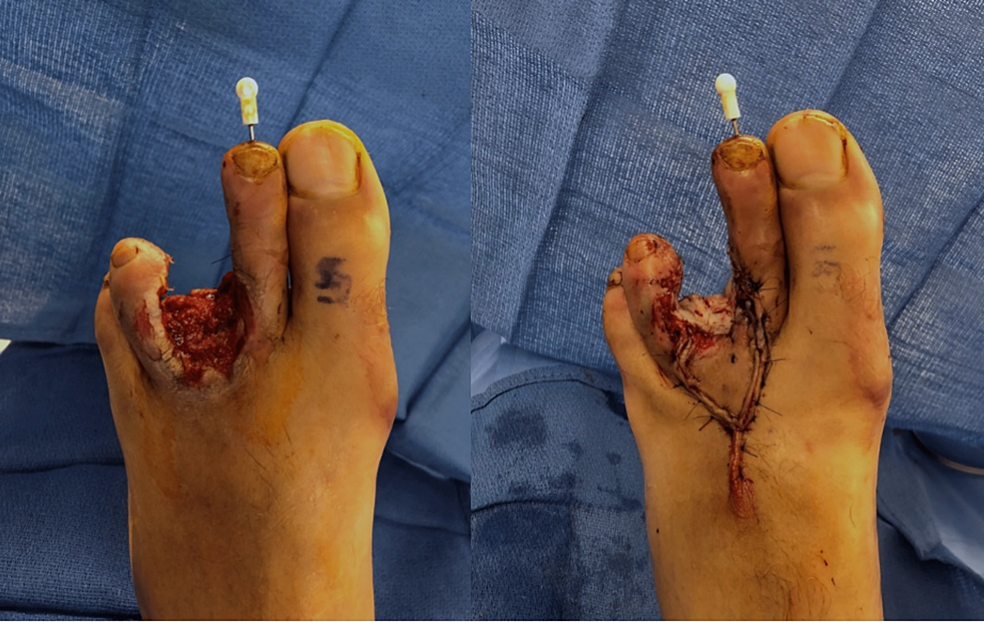
Gunshot wound to the third toe with amputation, tissue rearrangement (dorsal V-Y advancement flap), and a full-thickness skin graft The figure is provided by the authors from our patient population. Informed consent has been obtained for the usage of the image.

Soft tissue defects can be large, particularly with high-velocity firearm injuries that cast a wide zone of injury or shotgun injuries with pellets dispersing over a wide area. Following thorough, often serial debridement(s), soft tissue reconstruction is undertaken and frequently requires the expertise of a reconstructive plastic surgeon. Coverage options include skin grafts, local or regional flaps, or free tissue transfer (Figure 3). Similar principles can be applied to lower extremity injuries as well (Figure 4).

While limb salvage is attempted when possible, there are instances where amputation may become necessary, particularly in the case of a lower extremity with a vascular injury, which has been associated with higher rates of morbidity and mortality, including high rates of amputation, when compared to other forms of penetrating injury [23,24]. Targeted muscle reinnervation (TMR) is a technique used by plastic and reconstructive surgeons to address residual leg pain (RLP) and phantom limb pain (PLP) among patients with amputated extremities. In this procedure, residual nerves from amputated limbs are transferred to reinnervate new muscle targets that have otherwise lost function. This has been shown to mitigate PLP in patients with neuromas and PLP suffering from chronic pain and can even be performed preemptively in patients undergoing planned amputation [25-27].

Head and neck reconstruction

Reconstructive techniques in the head and neck have improved drastically since the initial origins of facial reconstruction following firearm injury during World War I, with numerous reconstructive techniques described in the literature. Firearm injuries in this region are seen in cases of assault, attempted suicide, and accidental injury. They carry a higher rate of mortality when compared to bodily firearm traumas [28,29].

Initial management is focused on first stabilizing the patient with a primary survey, paying special attention to the management of airways, and ruling out cervical spine injuries. In cases where endotracheal airways are difficult to maintain due to diffuse bleeding or disruption of facial structures, tracheostomies can be performed [30]. While surgery is not always needed, injuries involving the teeth, oral cavity, mandible, and comminuted fractures have a higher likelihood of requiring surgery [31]. Reconstructive efforts include immediate debridement and skeletal fixation, followed by free tissue transfer for any soft tissue coverage. Revisions can be performed at a later date for an improved aesthetic outcome [32].

**Figure 5:**
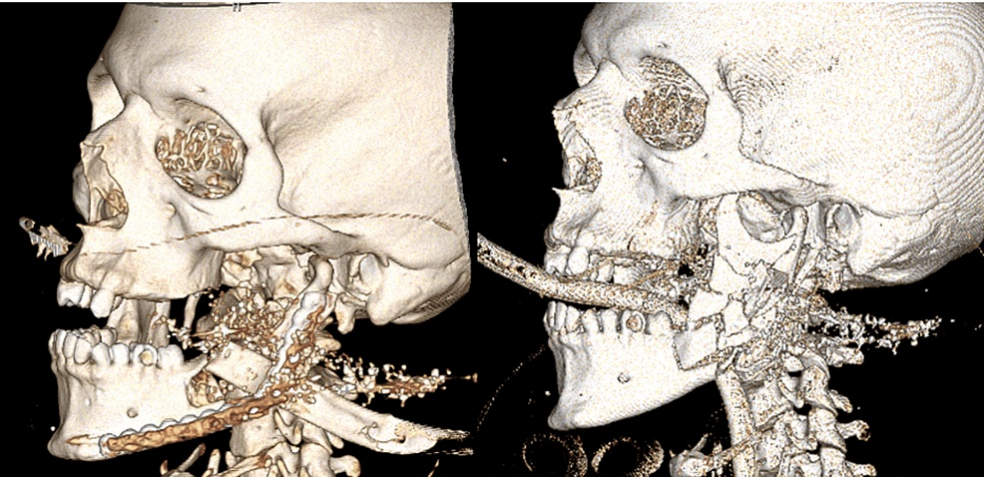
Mandiblular gunshot wound with mandibular reconstructive bar plate. The figure is provided by the authors from our patient population. Informed consent has been obtained for the usage of the image.

Due to the extensive risk of injury to both bone and soft tissue, reconstruction in this area presents a greater challenge than in the limbs. Primary closure is used for any superficial lacerations, while skin and myocutaneous flaps [33], including those from the pectoralis muscle, offer options for defect coverage in this region. For bony injuries, including Le Fort fractures, infraorbital fractures, and injuries to the mandible, open reduction and internal fixation (ORIF) are used (Figure 5). Maxillomandibular fixation (MMF) with the application of hybrid or Erich arch bars stabilizes fractures of the mandible and maxilla and can be performed by plastic surgeons, oral and maxillofacial surgeons, as well as otolaryngologists. Several delayed surgeries by plastic surgeons or these other surgical services may be necessary for complicated injuries in the head and neck region, particularly if speech or swallowing are affected [31].

For reconstruction of bony fragments of the mandible, free tissue transfer can be used, with free flaps from the fibula, scapular ocutaneous, and osteocutaneous radial forearm being most commonly used [34]. Ocular injuries merit an ophthalmology consult to prevent long-term issues with vision, but studies show that visual issues can occur nearly 44% of the time [35]. For orbital floor fractures, a repair can be performed by trained surgeons via the insertion of a mesh plate to prevent downward displacement of the globe [36].

Future directives and impact

Surgeon-driven initiatives, such as STOP THE BLEED, are aimed at educating the general public on how to prevent massive blood loss during acute firearm injuries; however, larger public health initiatives are needed to prevent firearm injuries on a larger scale. As community leaders, physicians and surgeons alike hold considerable power to help prevent firearm violence, with many physicians agreeing that firearm violence is a growing public health issue for which they can make a considerable impact [37,38].

Accidental injuries from firearms represent one area in which plastic surgeons can intervene. In addition to being the leading cause of pediatric mortality in the United States, accidental injury of the UE by firearms represents a majority of pediatric firearm-related morbidity as well [39]. Hand surgeons, in particular, are uniquely situated to address the ongoing prevention of firearm injuries through patient screening, counseling, and education regarding how to properly store firearms so they are out of reach from minors in the home and how to safely clean and unload or load firearms by first ensuring safety measures are in place. While former legislation in the state of Florida prohibited physicians from discussing firearm ownership with patients, with fines of nearly $10,000 and threats of losing one’s medical license, these policies were disbanded as discussing firearm safety was considered harm reduction and encouraged by the American Medical Association (AMA) [40].

Advocacy represents another domain through which plastic surgeons can raise awareness about firearm injury. Similar efforts have been made by plastic surgeons to raise awareness around firework-related ballistic injuries during the Fourth of July [41], as nearly 15,600 firework-related injuries were treated in emergency departments in the United States in 2020 [42], with many of these injuries involving the hand [43]. Suicide prevention is another area in which physicians play an important role, as access to a firearm is considered a risk factor for completing suicide. Simply asking patients about firearm ownership can help screen for persons at risk for suicide [44].

Furthermore, as there is a higher density of firearm injuries concentrated in large urban settings, particularly among communities of lower socioeconomic status, lower education, lower income, and higher unemployment rates [2], these interventions spearheaded by physicians have the potential to make a considerable impact on the community and reduce firearm violence disparities. Particularly in large urban centers with level-one trauma centers, knowledge of how to properly counsel patients regarding firearm safety is important.

## Conclusions

Acute surgical management of firearm injuries is critical for reducing firearm mortality. Reconstruction by plastic surgeons following firearm injury can help restore form and function, thereby limiting firearm morbidity and mortality. Operative techniques used by plastic surgeons include wound closure, skin grafting, flap reconstruction, repair of blood vessels, tendons, and/or nerves, fixation of fractures, and targeted muscle reinnervation in cases where limb salvage is not possible.

In addition to reconstructive efforts, the public health implications for plastic surgeons treating firearm-related injuries are high and include raising awareness among patients who are firearm owners regarding proper firearm storage as well as reinforcing proper cleaning techniques, including safe unloading of the firearm prior to cleaning. The potential for future impact among plastic surgeons and the community also remains high through advocacy and raising community awareness of the impact of firearm violence and associated morbidities. As firearm violence continues to evolve, plastic surgeons will play a considerable role in restoring form and function among patients afflicted with GSWs and counseling patients who are firearm owners regarding firearm safety.

## References

[1] Pino EC, Gebo E, Dugan E, Jay J (2022). Trends in violent penetrating injuries during the first year of the COVID-19 pandemic. JAMA Netw Open.

[2] Van Dyke ME, Chen MS, Sheppard M (2022). County-level social vulnerability and emergency department visits for firearm injuries - 10 U.S. jurisdictions, January 1, 2018-December 31, 2021. MMWR Morb Mortal Wkly Rep.

[3] (2023). Preventing gun violence, the leading cause of childhood death. https://www.nichd.nih.gov/about/org/od/directors_corner/prev_updates/gun-violence-July2022.

[4] (2023). Firearms are the leading cause of death for children in the United States but rank no higher than fifth in other industrialized nations. https://www.kff.org/private-insurance/press-release/firearms-are-the-leading-cause-of-death-for-children-in-the-united-states-but-rank-no-higher-than-fifth-in-other-industrialized-nations/.

[5] (2022). AMA calls gun violence "a public health crisis". https://www.ama-assn.org/press-center/press-releases/ama-calls-gun-violence-public-health-crisis.

[6] Lei R, Swartz MD, Harvin JA, Cotton BA, Holcomb JB, Wade CE, Adams SD (2019). Stop the Bleed Training empowers learners to act to prevent unnecessary hemorrhagic death. Am J Surg.

[7] Stathopoulos P (2018). Maxillofacial surgery: the impact of the Great War on both sides of the trenches. Oral Maxillofac Surg.

[8] (2022). The birth of plastic surgery. https://www.nam.ac.uk/explore/birth-plastic-surgery.

[9] Whitaker IS, Karoo RO, Spyrou G, Fenton OM (2007). The birth of plastic surgery: the story of nasal reconstruction from the Edwin Smith Papyrus to the twenty-first century. Plast Reconstr Surg.

[10] Al-Benna S, Bruce-Chwatt A, Gohritz A (2020). The origins of modern plastic surgery. J Plast Reconstr Aesthet Surg.

[11] Zhang WY, Hallock GG (2020). Gillies and Dunedin: the birthplace of modern plastic surgery. J Plast Reconstr Aesthet Surg.

[12] Manahan MA, Milner SM (2018). The Gillies's approach to posttraumatic reconstruction of ballistic injuries in evidence a century later. Eplasty.

[13] Gotsch KE, Annest JL, Mercy JA, & Ryan GW (2001). Surveillance for fatal and nonfatal firearm-related injuries — United States, 1993-1998. MMWR Surveill.

[14] Tarkunde YR, Clohisy CJ, Calfee RP, Halverson SJ, Wall LB (2021). Firearm injuries to the wrist and hand in children and adults: an epidemiologic study. Hand (N Y).

[15] Dougherty PJ, Vaidya R, Silverton C, Bartlett C, Najibi S (2009). Joint and long-bone gunshot injuries. J Bone Joint Surg Am.

[16] Sinauer N, Annest JL, Mercy JA (1996). Unintentional, nonfatal firearm-related injuries. A preventable public health burden. JAMA.

[17] Leong SC (2006). Unusual hand injury from cleaning a gun. Eur J Orthop Surg Traumatol.

[18] Miller M, Azrael D, Hemenway D, Vriniotis M (2005). Firearm storage practices and rates of unintentional firearm deaths in the United States. Accid Anal Prev.

[19] Ignatiadis IA, Mavrogenis AF, Igoumenou VG, Polyzois VD, Tsiampa VA, Arapoglou DK, Spyridonos S (2019). Gunshot and blast injuries of the extremities: a review of 45 cases. Eur J Orthop Surg Traumatol.

[20] Meade A, Hembd A, Cho MJ, Zhang AY (2021). Surgical treatment of upper extremity gunshot injures: an updated review. Ann Plast Surg.

[21] Cable DG, Mullany CJ, Schaff HV (1999). The Allen test. Ann Thorac Surg.

[22] Ghareeb PA, Daly C, Liao A, Payne D (2018). Current trends in the management of ballistic fractures of the hand and wrist: experiences of a high-volume level I trauma center. Hand (N Y).

[23] Siracuse JJ, Farber A, Cheng TW, Jones DW, Kalesan B (2020). Lower extremity vascular injuries caused by firearms have a higher risk of amputation and death compared with non-firearm penetrating trauma. J Vasc Surg.

[24] Siracuse JJ, Cheng TW, Farber A (2019). Vascular repair after firearm injury is associated with increased morbidity and mortality. J Vasc Surg.

[25] Dumanian GA, Potter BK, Mioton LM (2019). Targeted muscle reinnervation treats neuroma and phantom pain in major limb amputees: a randomized clinical trial. Ann Surg.

[26] Valerio IL, Dumanian GA, Jordan SW (2019). Preemptive treatment of phantom and residual limb pain with targeted muscle reinnervation at the time of major limb amputation. J Am Coll Surg.

[27] Mioton LM, Dumanian GA, Shah N (2020). Targeted muscle reinnervation improves residual limb pain, phantom limb pain, and limb function: a prospective study of 33 major limb amputees. Clin Orthop Relat Res.

[28] Yuksel F, Celikoz B, Ergun O, Peker F, Açikel C, Ebrinc S (2004). Management of maxillofacial problems in self-inflicted rifle wounds. Ann Plast Surg.

[29] Doctor VS, Farwell DG (2007). Gunshot wounds to the head and neck. Curr Opin Otolaryngol Head Neck Surg.

[30] Hollier L, Grantcharova EP, Kattash M (2001). Facial gunshot wounds: a 4-year experience. J Oral Maxillofac Surg.

[31] Kothamasu VS, Deramo P, Biaggi-Ondina AP, Kim BW, Wainwright DJ (2020). Surgical management of gunshot wounds to the face. Plast Reconstr Surg Glob Open.

[32] Vaca EE, Bellamy JL, Sinno S, Rodriguez ED (2018). Management of high-energy avulsive ballistic facial injury: a review of the literature and algorithmic approach. Plast Reconstr Surg Glob Open.

[33] Aukerman W, Hull M, Nannapaneni S, Shayesteh K (2021). Facial gunshot wound: mandibular fracture with internal fixation and a pectoralis myocutaneous flap coverage. Cureus.

[34] Gurunluoglu R, Gatherwright J (2019). Microsurgical reconstruction of complex maxillofacial gunshot wounds: outcomes analysis and algorithm. Microsurgery.

[35] Chopra N, Gervasio KA, Kalosza B, Wu AY (2018). Gun trauma and ophthalmic outcomes. Eye (Lond).

[36] Momeni Roochi M, Razmara F (2020). Maxillofacial gunshot injures and their therapeutic challenges: case series. Clin Case Rep.

[37] Cassel CK, Nelson EA, Smith TW, Schwab CW, Barlow B, Gary NE (1998). Internists' and surgeons' attitudes toward guns and firearm injury prevention. Ann Intern Med.

[38] Wintemute GJ, Betz ME, Ranney ML (2016). Yes, you can: physicians, patients, and firearms. Ann Intern Med.

[39] Nichols DS, Audate M, King C, Kerekes D, Chim H, Satteson E (2021). Pediatric upper extremity firearm injuries: an analysis of demographic factors and recurring mechanisms of injury. World J Pediatr.

[40] (2022). Court strikes down Florida law barring doctors from discussing guns with patients. https://www.npr.org/sections/thetwo-way/2017/02/17/515764335/court-strikes-down-florida-law-barring-doctors-from-discussing-guns-with-patient.

[41] (2022). KSHB: plastic surgeon warns of firework hand injuries. https://www.saintlukeskc.org/about/news/kshb-plastic-surgeon-warns-firework-hand-injuries.

[42] (2022). Fireworks-related injuries and deaths spiked during the COVID-19 pandemic. https://www.cpsc.gov/Newsroom/News-Releases/2021/Fireworks-Related-Injuries-and-Deaths-Spiked-During-the-COVID-19-Pandemic.

[43] Ortiz R, Ozkan S, Chen NC, Eberlin KR (2020). Firework injuries of the hand: an analysis of treatment and health care utilization. Hand (N Y).

[44] Richards JE, Boggs JM, Rowhani-Rahbar A, Kuo E, Betz ME, Bobb JF, Simon GE (2022). Patient-reported firearm access prior to suicide death. JAMA Netw Open.

